# Cardiogenol C can induce Mouse Hair Bulge Progenitor Cells to Transdifferentiate into Cardiomyocyte-like Cells

**DOI:** 10.1186/1477-5956-9-3

**Published:** 2011-01-19

**Authors:** Winifred WY Yau, Mei Kuen Tang, Elve Chen, YaoYao, Ivan WC Wong, Henry SS Lee, Kenneth KH Lee

**Affiliations:** 1 Stem Cell and Regeneration Thematic Research Programme, School of Biomedical Sciences, Chinese University of Hong Kong, Shatin, Hong Kong; 2 Key Laboratory for Regenerative Medicine, Ministry of Education, China

## Abstract

**Background:**

Hair bulge progenitor cells (HBPCs) are multipotent stem cells derived from the bulge region of mice vibrissal hairs. The purified HBPCs express CD34, K15 and K14 surface markers. It has been reported that HBPCs could be readily induced to transdifferentiate into adipocytes and osteocytes. However, the ability of HBPCs to transdifferentiate into cardiomyocytes has not yet been investigated.

**Methodology/Principal Findings:**

The cardiomyogenic potential of HBPCs was investigated using a small cell-permeable molecule called Cardiogenol C. We established that Cardiogenol C could induce HBPCs to express transcription factors GATA4, Nkx2.5 and Tbx5, which are early specific markers for pre-cardiomyogenic cells. In prolonged cultures, the Cardiogenol C-treated HBPCs can also express muscle proteins, cardiac-specific troponin I and sarcomeric myosin heavy chain. However, we did not observe the ability of these cells to functionally contract. Hence, we called these cells cardiomyocyte-like cells rather than cardiomyocytes. We tried to remedy this deficiency by pre-treating HBPCs with Valproic acid first before exposing them to Cardiogenol C. This pretreatment inhibited, rather than improved, the effectiveness of Cardiogenol C in reprogramming the HBPCs. We used comparative proteomics to determine how Cardiogenol C worked by identifying proteins that were differentially expressed. We identified proteins that were involved in promoting cell differentiation, cardiomyocyte development and for the normal function of striated muscles. From those differentially expressed proteins, we further propose that Cardiogenol C might exert its effect by activating the Wnt signaling pathway through the suppression of Kremen1. In addition, by up-regulating the expression of chromatin remodeling proteins, SIK1 and Smarce1 would initiate cardiac differentiation.

**Conclusions/Significance:**

In conclusion, our CD34^+^/K15^+ ^HBPCs could be induced to transdifferentiate into cardiomyocyte-like cells using a small molecule called Cardiogenol C. The process involves activation of the Wnt signaling pathway and altered expression of several key chromatin remodeling proteins. The finding is clinically significant as HBPCs offer a readily accessible and autologous source of progenitor cells for cell-based therapy of heart disease, which is one of major killers in developed countries.

## Introduction

The hair follicle is a structure that constantly undergoes cyclic self-renewal of anagen (growth), catagen (regression) and telogen (resting) stages for the replacement of natural hair loss [1]. Studies over the past two decades have been documented the presence of a progenitor cell population residing in the hair bulge region, near where the arrector pili muscle attaches to the outer hair root sheath [2,3]. It was elucidated that hair bulge progenitor cells (HBPCs) were derived from neural crest cells that migrated to the bulge during embryonic development [4,5]. These neural crest cells that are multipotent have the capability to differentiate into various cell types in the embryo, including neurons, schwann cells, glial cells, sensory neurons, melanocytes, endocrine cells, chondrocytes and smooth muscles [5-9]. It has been reported that there are cardiac neural crest-derived cells residing in the heart, as a rare population of dormant multipotent stem cells that can be induced to differentiate into cardiomyocytes when given the appropriate stimulation [10]. However, it would be impractical to harvest cardiac neural crest cells as a source of progenitor cells for the therapeutic repair of damaged heart tissues. Therefore, it is useful to identify a reservoir of these progenitor cells, which are abundant and readily accessible. HBPCs are readily accessible since they reside on the outer root sheath of the hair follicle and contain a rich source of neural crest-derived progenitor cells, but their ability to transdifferentiate into cardiomyocytes has never been investigated. In this context, it is important to establish a method for directing HBPCs to transdifferentiate into cardiomyocytes. There are several known chemicals that can induce embryonic and bone marrow-derived mesenchymal stem cells into cardiomyocytes-like cells, such as dimethyl sulfoxide and 5-azacytidine [11-17]. Although the induction mechanisms are not yet fully understood, it has been reported that the structure of 5-azacytidine is similar to cytidine. 5-azacytidine can induce demethylation of cytosine and activate the expression of myogenic gene MyoD1 which in turn facilitates the differentiation of bone marrow stem cells into cardiomyocyte-like cells [16]. Wu et al. synthesized a novel small molecule from a class of diaminopyrimidine compounds, called Cardiogenol C that could specifically induce embryonic stem cells to differentiate into the cardiomyocytes [18]. They reported that up to 90% of the Cardiogenol C treated cells positively expressed GATA4, Mef2 and Nkx2.5, which are essential transcription factors involved in cardiogenesis. To date, Cardiogenol C has not been applied to induce adult stem cells type to differentiate into cardiomyocytes. Moreover, it is still not known how this molecule works or the proteins that it targets.

In the present study, we first investigated the multipotency of HBPCs and then tested the ability of Cardiogenol C to induce HBPCs to transdifferentiate into cardiomyocytes. In addition, we used comparative proteomics to understand how Cardiogenol C worked by identifying differentially expressed proteins that were directly or indirectly influenced by Cardiogenol C.

## Materials and methods

### Ethics Statement

All experimental procedures have been approved by the animal ethics committee, The Chinese University of Hong Kong with approval number (09-245) in DH/HA&P/8/2/1 Pt.7.

### Isolation of hair bulge explants

**Figure 1:**
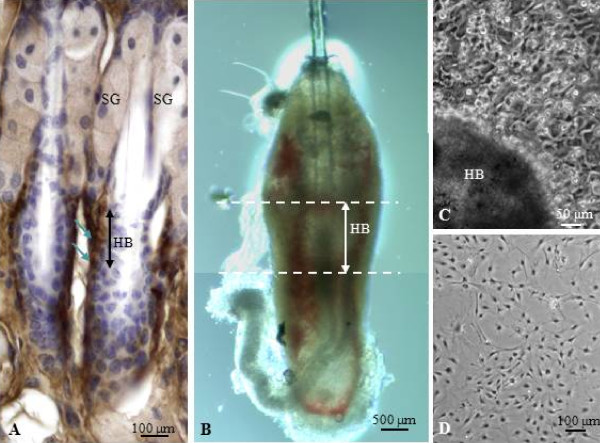
**Establishment of primary hair bulge cell culture**. (A) Immunohistological staining revealed a large number of CD34^+ ^cells (arrows) present in the vicinity of the hair bulge (HB) inferior to sebaceous glands (SG). (B) Showing an isolated hair follicle from mouse vibrissal hair. The hair bulge was dissected (extent indicated by the dotted lines) from the follicle and used for explant culture. (C) Showing cells that have migrated out from the bulge explant to form colonies of cells all around the explant. (D) Showing the appearance of HBPCs following purification with anti-CD34 conjugated magnetic beads.

Adult female ICR mice (8-10 weeks old) were sacrificed by cervical dislocation and anagen staged vibrissal hair follicles were extracted from the whisker pads according to methods reported by Sieber-Blum et al. [5]. Briefly, the whisker pads were isolated and sterilized in 70% ethanol for 1 min and then washed 3 times in dissecting medium (DMEM/F12, 0.5% FBS, Invitrogen). Under the dissecting microscope, the dermis and adipose tissues were carefully removed from the vibrissal hair follicle using sharp tungsten needles. The follicle was then cut at cross-sectioned at levels above the cavernous sinus and below the attachment for the arrestor pili muscle (Figure 1B). After the hair bulge region was isolated, it was then plated onto a collagen-coated 35-mm organ culture dish containing 0.5 ml culture medium. The culture medium is composed of the Glasgow Minimal Essential Medium (GMEM; Sigma-Aldrich, St. Louis, USA), supplemented with 10% USDA-approved embryonic stem-cell qualified fetal bovine serum (ESQ-FBS, Invitrogen), and penicillin-streptomycin (0.01 mg/ml). The explants were maintained in 5% CO_2 _at 37 °C inside a humidified cell incubator. The culture medium was changed every three days.

### Production, isolation and purification of CD34^+ ^HBPCs

After seven days culture, cells have migrated out from all around the hair bulge explant (Figure 1C). The explant was then removed using the tungsten needles and the cells that have attached to the culture plate were rinsed with PBS and digested with 0.25% trypsin solution (0.03% EDTA, 10 mM HEPES in Hank's balanced salt solution) for 2 min. The reaction was then stopped with GMEM plus 1% ESQ-FBS and the cell suspension was further centrifuged at 1,500 rpm for 3 min. These cells were resuspended and seeded onto two 60-mm culture dishes in GMEM with 10% ESQ-FBS, 5% CO_2 _at 37°C inside the humidified cell incubator. It has been reported the HBPCs expressed cell surface marker CD34, therefore we employed Dynal CD34 Progenitor Cell Selection System (Invitrogen) to select CD34^+ ^HBPCs out from our cell cultures. Briefly, 4 × 10^7^/100 μl of CD34-coated magnetic beads (Dynabeads, Invitrogen) were first washed with 1 ml of isolation buffer (PBS without Ca^2+ ^and Mg^2+^, 0.1% BSA and 2 mM EDTA, pH 7.4). The tube was placed in a magnetic stand (Dynal MPC-S Magnetic Particle Concentrator; Invitrogen) and then the supernatant was aspirated. The tube was then removed from the magnetic stand, and the washed magnetic beads resuspended in 100 μl of isolation buffer, ready for use. The primary hair bulge cultures were trypsinized and the cells were suspended at 1 × 10^8 ^cells/ml. The appropriated cell density of 1 ml of the crude hair bulge cells suspension was mixed with 100 μl of pre-washed magnetic beads. The mixture was then incubated at 4°C for 30 min with gentle tilting and rotation. The tube was then filled with isolation buffer (to the level of the magnets) and the cell-bead complexes were resuspended. The tube was placed in the magnetic stand for 2 min and then the supernatant was discarded. The bead-bound cells were washed and resuspended in 100 μl of isolation buffer. The suspension was further centrifuged for 10 min at 400 × g to remove excess detached beads. Finally, the purified CD34^+ ^HBPCs pellet was resuspended and cultured in GMEM plus 10% ESQ-FBS.

### Testing the multipotency of the CD34^+ ^HBPCs

CD34^+ ^HBPCs were assessed for their ability to transdifferentiate into adipocytes, osteocytes and cardiomyocytes. Purified HBPCs (1 × 10^5 ^cells/ml), in normal culture medium, were plated onto four-well culture plates containing 13-mm glass coverslips. After incubation at 37°C overnight, the HBPCs were treated with adipogenic inducing medium composing of GMEM, 1 mg/ml insulin (Sigma-Aldrich), 100 μM dexamethasone (Sigma-Aldrich), 100 mM 3-isobutyl-1-methylxanthine (Sigma-Aldrich) and 7.5% ESQ-FBS. After three weeks culture, the presence of adipocytes was determined using Oil Red O staining. For osteogenic induction, we used medium containing GMEM, 10 mM β-glycerophosphate (Sigma-Aldrich), 50 μM ascorbic acid 2-phosphate, 1 μM dexamethasone and 7.5% ESQ-FBS. After 3-weeks culture, the presence of osteocytes was identified using Alizarin Red S staining, which detected the presence of mineralized calcium-deposits. For cardiogenic induction, we used GMEM plus 5 μM Cardiogenol C (Calbiochem, CA, USA) and 7.5% ESQ-FBS. The cultures were harvested at different day-intervals after induction for immunohistochemistry, semi-quantitative RT-PCR analysis, western blot analysis and comparative proteomic.

### Immunohistochemistry

Briefly, Cardiogenol C treated and untreated CD34^+ ^HBPCs that have been cultured on coverslips were fixed in 10% formalin overnight. The samples washed 3 times with PBS and permeabilized with 2 M HCl with 0.5% (v/v) Triton X-100 for 30 min. These samples were then blocked with 3% BSA (Amresco) in PBS for 1 hr, and incubated with primary antibody (1:100) overnight at room temperature with gentle agitation. Primary antibodies used were mouse monoclonal antibodies against CD34 (Lab Vision, CA, USA), K14 (Calbiochem, CA, USA), active β-catenin (Millipore, CA, USA), GATA4 (Santa Cruz Biotechnology, CA, USA), sarcomeric myosin heavy chain (Developmental Studies Hybridoma Bank, Iowa, IA, USA), Cardiac-specific troponin I (Lab Vision) and Islet1 (R & D Systems). In addition, rabbit monoclonal anti-K15 (Epitomics, CA, USA) and goat polyclonal anti-Nkx-2.5 antibodies (Santa Cruz Biotechnology, CA, USA) were also used. The cells were washed three times with PBST (PBS buffer with 0.05% Tween 20) for 20 min to remove unbound primary antibody. Afterwards, the appropriate secondary antibody (1:300) was added for 1 hr at room temperature in the dark with gentle shaking. The secondary antibodies used were FITC-conjugated donkey anti-mouse immunoglobulin G (IgG) and Cy2-conjugated donkey anti-goat IgG (Jackson ImmunoResearch). Unbound secondary antibody was removed by washing with PBST and then PBS. The samples were counterstained with the nuclear stained dye DAPI (Molecular Probes, Eugene, OR, USA) in 50% (v/v) glycerol and mounted onto slides. The samples were then examined and recorded under a confocal microscopy (Confocal Laser Scanning Biological Microscope FV1000; Olympus, Japan) with fixed exposure settings for all the samples. Image analysis was performed using a FV10-ASW software (version 1.6a, Olympus). Three replicates of each sample were analyzed.

### Semi-quantitative RT-PCR analysis

Total RNA was isolated from Cardiogenol C treated and untreated CD34^+ ^HBPCs using TRIzol Reagent (Invitrogen Life Technologies). First strand cDNA was synthesized using Ready-To-Go You-Prime First-Strand Beads (GE Healthcare), according to manufacturer's instructions. PCR was performed using 1 μl of the synthesized cDNA as the template, 2.5 μl of 10× PCR buffer (Bio-Firm, Hong Kong), 1 μl of 50 mM magnesium chloride solution (Bio-Firm, Hong Kong), 5 μl of 2 mM dNTP mix (Bio-Firm, Hong Kong), 1 unit of β Taq DNA polymerase (Bio-Firm, Hong Kong), 1 μl of forward and reverse primers (10 μM; 1^st ^BASE, Singapore), and DEPC-treated water was added up to a final volume of 25 μl. The primers, listed in Table 1 were designed using Primer3 software (version 0.4.0, Rozen and Skaletsky; http://frodo.wi.mit.edu/ April 13, 2008). The reaction mixture was then placed in a PTC-100 thermal cycler with a heated lid (MJ Research, Watertown, MA, USA) operated under the following amplification conditions: initial denaturation at 95°C for 2 min, followed by a total of 35 cycles of denaturation at 95°C for 1 min, annealing at 55°C for 1 min, and extension at 72°C for 1 min. There was a final extension at 72°C for 5 min. The PCR products were analyzed by 1.5% agarose (USB) gel electrophoresis and stained with ethidium bromide (0.5 μg/ml). The expected bands in the gels were then examined under ultraviolet (UV) light, using a FluorChem 8000 imaging system (Alpha Innotech, San Leandro, CA). The intensity of the band for each sample was normalized using housekeeping β-actin gene. Each experiment was repeated three times.

**Table 1:** Primers for semi-quantitative RT-PCR analysis

Gene		Primer sequences (5' to 3')	Product size (bp)	
β-actin	Sense	5'-TGT TAC CAA CTG GGA CGA C-3'	573	
	Anti-sense	5'-AAG GAA GGC TGG AAA AGA G-3'		

CD34	Sense	5'-TAG CCC AGT CTG AGG TTA GG-3'	205	
	Anti-sense	5'-AGC AGA ACT CCA GAG GTG AC-3'		

Dkk1	Sense	5'-TGT CTA GGG GGT CGA ATG TA-3'	300	
	Anti-sense	5'-CAC AGA GCC CCA AAT GTA TC-3'		

Etv6	Sense	5'-GTC TCC TTC AGC AGA AAC CA-3'	424	
	Anti-sense	5'-CTG ATC CCA ACT GTG TGT CA-3'		

GATA4	Sense	5'-GTG GTG GGT TTT TCT TTG TG-3'	443	
	Anti-sense	5'-CAT AGT CAC CAA GGC TGC TT-3'		

Hes6	Sense	5'-AGG GTA GCA GCT TTC AGG AT-3'	354	
	Anti-sense	5'-CCT GAG CTG TCT CCA CCT TA-3'		

Keratin 5	Sense	5'-GGA CCA GTC AAC ATC TCT GT-3'	378	
	Anti-sense	5'-TGC CTA AAA GAA GCA GTC TC-3'		

Keratin 14	Sense	5'-GAA TGG TTC TTC AGC AAG AC-3'	386	
	Anti-sense	5'-ATG AGG AGA ATT GGG AAG AT-3'		

Kremen1	Sense	5'-GCA TCC ATT TCA ACT TCA CC-3'	431	
	Anti-sense	5'-CGT ACA CAG TCC ATC CTT CC-3'		

Mthfr	Sense	5'-CGT AGA GCA ACT TAG GGA GA-3'	314	
	Anti-sense	5'-CCC ATG AGA AGA ACT AGC AG-3'		

Nestin	Sense	5'-GAA TCA GAT CGC TCA GAT CC-3'	487	
	Anti-sense	5'-GCA CGA CAC CAG TAG AAC TGG-3'		

Nkx-2.5	Sense	5'-ATC CTA AAC CTG GAG CAG CA-3'	501	
	Anti-sense	5'-AGA TCT TGA CCT GCG TGG AC-3'		

Osteocalcin	Sense	5'-GCA GCT TGG TGC ACA CCT AG-3'	405	
	Anti-sense	5'-ACC TTA TTG CCC TCC TGC TT-3'		

Phc1	Sense	5'-AGC AGA CTT TGC AGA GAA GA-3'	212	
	Anti-sense	5'-TAT ACT GGC TGG GGA TCC TG-3'		

Plod2	Sense	5'-GCC AGA AGG TGA GAT TAC TG-3'	397	
	Anti-sense	5'GCA CAA CAG GGT ACT TGT CT-3'		

PPAR-γ	Sense	5'-ACT GTC GGT TTC AGA AGT GC-3'	258	
	Anti-sense	5'-GAT CTC TTG CAC GGC TTC TA-3'		

Sik1	Sense	5'-GGA GAG GAA AGG AGA CAC TT-3'	296	
	Anti-sense	5'-GGA TAC TGA GTG CTT GCT TC-3'		

Sox2	Sense	5'-GCC CTG CAG TAC AAC TCC AT-3'	363	
	Anti-sense	5'-GTC ATT TGC TGT GGG TGA TG-3'		

Srms	Sense	5'-TCC CTA TGA AGG AAT GAC CA-3'	354	
	Anti-sense	5'-AGC CTG TGT CAG TGA AGG AG-3'		

Tbx5	Sense	5'-CAA AGA CAG GTC TTG CGA TT-3'	426	
	Anti-sense	5'-GGT GAG TTT GAG CTT CTG GA-3'		

Tcf3	Sense	5'-CTT ATC CCC CAA CTA CGA TG-3'	315	
	Anti-sense	5'-ATA GGA GTC GGG AGG TCT CT-3'		

Wnt11	Sense	5'-AGG ATC CCA AGC CAA TAA AC-3'	330	
	Anti-sense	5'-GGA TCC CAC CTT CTC ATT CT-3'		

YY1	Sense	5'-GCG GCA AGA AGA GTT ACC TG-3'	418	
	Anti-sense	5'-GGT GTG CAG ATG CTT TCT CA-3'		

Ezh2	Sense	5'-AGG CTA ATT GGG ACC AAA AC-3'	306	
	Anti-sense	5'-AAA GCG GTT TTG ACA CTC TG-3'		

### Western blot analysis

Briefly, Cardiogenol C treated and untreated of HBPCs were trypsinized and harvested by centrifugation at 1,500 rpm for 3 min. The protein lysate samples were then prepared from the cell pellets that were solubilized in 200 μl of lysis buffer made up of 8 M urea, 2% CHAPS (Sigma-Aldrich), 2 M thiourea (Sigma-Aldrich), 40 mM dithiothreitol (Sigma-Aldrich), 1% (v/v) Nonidet P-40 (USB, USA), 0.01% (v/v) TBP, Bezonase^® ^nuclease (Novagen, USA) and a cocktail of protease inhibitors (consisting of leupeptin, pepstatin, aprotinin, PMSF and EDTA-Na_2_; Roche Molecular Biochemicals, USA). After incubation on ice for 2 hr, the cell lysate samples were centrifuged at 12,000 rpm at 4°C for 15 min. The supernatant, which contained the proteins, was transferred into Eppendorf tubes. The concentration of protein for each sample was determined using a Bio-Rad Protein Assay Kit. After SDS-PAGE, the proteins were transferred using a Trans-Blot Semi-Dry Transfer Cell (Bio-Rad) set at 90 mA for 60 min. The blots were stained with Ponseau S (Sigma-Aldrich) to confirm the presence of the proteins. The blots were then blocked with 5% skimmed milk and 1:1,000 primary antibodies added to the blots overnight at 4°C with agitation. Primary antibodies used were mouse monoclonal antibodies against β-catenin (Millipore, CA, USA), Ezh2 (Abnova), Kremen1 (Clone 252525, R&D Systems), Phc1 (Abnova) and tubulin-α (LabVision). The blots were then washed with TBST (TBS buffer with 0.05% Tween 20) and probed with the appropriate HRP-conjugated secondary antibody solution (1:10,000, Zymed), and incubated for 1 hr with gentle agitation. Finally, the blots were washed and developed using an ECL Western blotting detection kit (GE Healthcare), according to manufacturer's instructions. There were three replicates of each sample. The staining was viewed and analyzed using a FluorChem 8000 imaging system (Alpha Innotech Corporation, San Leandro, CA). The band intensity measurement for each protein band was recorded and normalized against measurements housekeeping protein tubulin-α. All procedures were performed in triplicate and results were expressed as the mean value.

### Cell proliferation assay

The effects of Cardiogenol C on HBPCs proliferation were determined by MTT assay. Briefly, 200 μl of CD34^+ ^HBPCs (1 × 10^4 ^cells/ml) was seeded into a 96-well plate. The cells were allowed to adhere and then treated with Cardiogenol C. At set time intervals between 1-5 days, 20 μl of 12 mM 3-(4,5-dimethylthiazol-2-yl)-2, 5-diphenyltetrazolium bromide (MTT) solution in medium without the phenol red was added to the cultures and incubated for 4 hr at 37°C. The supernatants were then discarded and 200 μl of DMSO solution was added. The plates were placed on an orbital shaker for 15 min to dissolve formazan crystals and then measured on a microplate reader (Bio-Rad) set at 490 nm. There were three replicates for each time point analyzed.

### Scanning electron microscopy

Briefly, treated and untreated HBPCs cultured on coverslips were washed with PBS and fixed in 2.5% glutaraldehyde dissolved in 0.1 M freshly prepared Sorensen's Phosphate Buffer (PB, pH 7.4) for 4 hr. The samples were post-fixed with 1% aqueous osmium tetraoxide for 15 min and washed 3 times in PB for 10 min. The samples were then dehydrated through a graded series of ethanol, critical point dried and coated with palladium-gold. The coated specimens were examined under a JSM-6301F scanning electron microscope (Joel, Tokyo, Japan).

### Transmission electron microscopy

The treated and untreated HBPCs were fixed in freshly prepared 2.5% glutaraldehyde in 0.1 M phosphate buffer (pH 7.4) for 4 h. After rinsing in phosphate buffer, the cells were post-fixed in 1% osmium tetraoxide for 30 min. The cultures were then washed with MilliQ-water, dehydrated through a graded series of ethanol, cleared in propylene oxide, and then embedded in Epon 812 embedding resin. The embedded cultures were sectioned with an UltraCut R microtome (Leica, Austria), double-stained with uranyl acetate and lead citrate, and then examined using a transmission electron microscope (Hitachi H-7100FA, Hitachi, Tokyo, Japan).

### Proteomics: sample preparations for two dimensional gel electrophoresis (2-DE)

Comparative proteomic analysis was performed as we previously reported [19,20]. Briefly, 100 μg of total proteins from Cardiogenol C treated and untreated CD34^+ ^HBPCs were used in each 2-DE. The samples were first washed in ice-cold saline and then lyzed in the presence of 7 M Urea, 2 M thiourea, 0.01% TBP, 4% CHAP, 0.01% NP 40 and a mixture of protease inhibitors (Roche Molecular Biochemicals, Mannheim, Germany). After 2 hr incubation at 4°C, the supernatants were harvested by centrifugation at 13,000 rpm for 15 min. The total protein concentration of the samples was determined using a protein assay kit (Bio-Rad, Hercules, USA).

### Proteomics: two dimensional gel electrophoresis

First dimensional separation of the proteins was performed on an IPGphor IEF system using immobiline pH 4 - 7 dry IPG strips (GE Healthcare, Uppsala, Sweden). The cell lysates (100 μg) were loaded onto rehydrated immobiline strips. The setting for step 1 was 500 volts for 500 vhr; step 2 was 1000 volts for 1000 vhr; step 3 at 2000 volts for 2000 vhr; step 4 at 3000 volts for 3000 vhr; step 5 at 4000 volts for 4000 vhr; step 6 at 5000 volts for 5000 vhr and finally, step 7 at 5600 volts for 20000 vhr. Vertical sodium dodecyl sulphate polyacrylamide gel electrophoresis (SDS-PAGE) was used for the second dimension, using 10% polyacrylamide slab gels (18 × 16 cm). Briefly, the gel strips were removed from the IPGphor IEF system and equilibrated for 30 min in 6 M urea, 30% w/v glycerol, 2% w/v SDS, 0.05 M Tris-HCl, pH 6.8 with 2% w/v DTT. They were then treated with 2% iodoacetamide for 30 min. The gel strips were embedded on the cathode side of a pre-prepared SDS-PAGE gel and 0.2% agarose was poured into the cathode side to seal the gel strip. The second dimension electrophoresis was performed in an ISO-DALT apparatus (Hoefer Scientific Instruments, San Francisco, USA). A tris-tricine dissociating buffer system was used and the gel was run at 60 mA constant current overnight. The gels were then fixed in 40% methanol containing 10% acetic acid for 1 hr and followed by a second fixative containing 50% ethanol. The fixed gels were further sensitized with 0.02% sodium thiosulphate for 10 min. After sensitization, the gels were stained with silver nitrate (Sigma-Aldrich Co., St Louis, USA) and developed. The molecular mass of the protein spots was determined by co-running the samples with standard protein markers, covering the range of 14.4 - 116 kDa (Fermanta International Inc, Burlington, Canada). The pI values were determined according to the information provided by the supplier of the IPG strips. The silver stained 2-DE gels of Cardiogenol C treated and untreated HBPCs were scanned using an Agfa DUOSCAN densitometer. The distribution of the protein spots in the 2-DE gels was recorded, compared and quantified using the ImageMaster 2 D Elite software (Amersham Biosciences, Freiburg, Germany). The data were normalized with respect to the total density of the gel image. Three replicates of each sample were analyzed.

### Proteomics: in-gel digestion and MALDI-TOF analysis

Protein spots were isolated from the silver stained gels using a spot picker (EXQuest™ Spot Cutter). Each isolated spot was destained in 500 μl of 15 mM potassium ferricyanide and 50 mM sodium thiosulfate for 10 min. The sample was then further washed 3 times for 15 min each in 500 μl of 50% acetonitrile/25 mM NH4 bicarbonate at pH 8.0. The spot was soaked in 100% acetonitrile for 5 min to dehydrate the gel, the acetonitrile was removed when the gel turned opaque white and the gel was finally dried in a Speed-Vac Evaporator (UniEquip, Martinsried, Germany). For enzyme digestion, the gel spot was rehydrated in cold trypsin (15 μg/mL, Promega, Madison) made up in 25 mM ammonium bicarbonate, pH 8.0. After the gel had swelled and cleared, it was incubated at 37°C for 16 - 24 h. The peptide was then extracted using 50% acetonitrile and 5% trifluoroacetic acid. The extract peptides (0.5 μl) were then mixed with 1 μl of fresh cyano-matrix solution (consisting of an 8 mg α-cyano-4-hydroxycinnamic acid in 50% acetonitrile/0.1% TFA) on a MALDI plate. The protein sample was analyzed in a time-of-flight mass spectrometer (Voyager-DE™PRO Biospectrometry™, Applied Biosystems) using an accelerating voltage of 20 kV. For database search, known contamination peaks such as autoproteolysis and keratin were extracted prior to a protein mass fingerprint search with MASCOT software (Matrix Science, London, UK) in CBInr database. Up to one missed tryptic cleavage was considered and a mass accuracy of 100 ppm was used for all tryptic-mass searches. Protein identification was confirmed by using MS-Fit software http://prospector.ucsf.edu.

## Results

### Isolation and Purification of CD34^+ ^HBPCs

**Figure 2:**
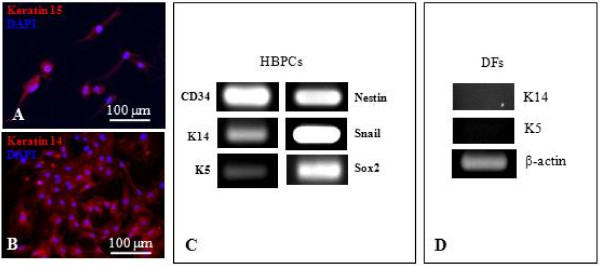
**Characterization of CD34^+ ^HBPCs**. (A-B) Immunofluorescent staining showed HBPCs specifically expressed established HBPCs surface markers Keratin 14 (K14) and 15 (K15). (C) Semi-quantitative RT-PCR analysis confirmed that the HBPCs expressed K14. In addition, HBPCs also expressed K5 and Snail and Sox2. (D) In contrast, dermal fibroblasts (DFs) isolated from adjacent to the hair bulge did not express K14 and K5. β-actin served as an internal control.

It has been reported that cell surface marker CD34 is specifically expressed by HBPCs isolated from the hair mouse bulge [21]. We performed immunohistological staining to determine where CD34^+ ^cells were normally distributed in the vibrissa. CD34^+ ^HBPCs were evident in the bulge region of the outer root hair sheath, inferior to the sebaceous glands (Figure 1A). We carefully microdissected and isolated the bulge area from the vibrissa follicles and explanted them onto organ culture dishes (Figure 1B). We observed cells migrating out from the bulge explants after seven days culture. Colonies of cells were found grown around the bulge region (Figure 1C) which were trypsinized and seeded onto the 60 mm plate (Figure 1D). The cells from the primary hair bulge culture was then harvested and purified using magnetic beads coated with CD34 monoclonal antibody. We also confirmed that these cells expressed other HBPC cell surface markers K15 and K14 (Figure 2A, B). Moreover, semi-quantitative RT-PCR revealed that these cells expressed K5, Snail, Sox2, K14, CD34 and Nestin (Figure 2C). Dermal fibroblasts, isolated from adjacent to the hair bulge, did not express any of the HBPC surface markers (Figure 2D). This confirms that our HBPCs were derived from cells that have migrated out from bulge explants and not from connective tissue cells that have contaminated the bulge explants during isolation.

### Establishing the multipotency of CD34^+ ^HBPCs

**Figure 3:**
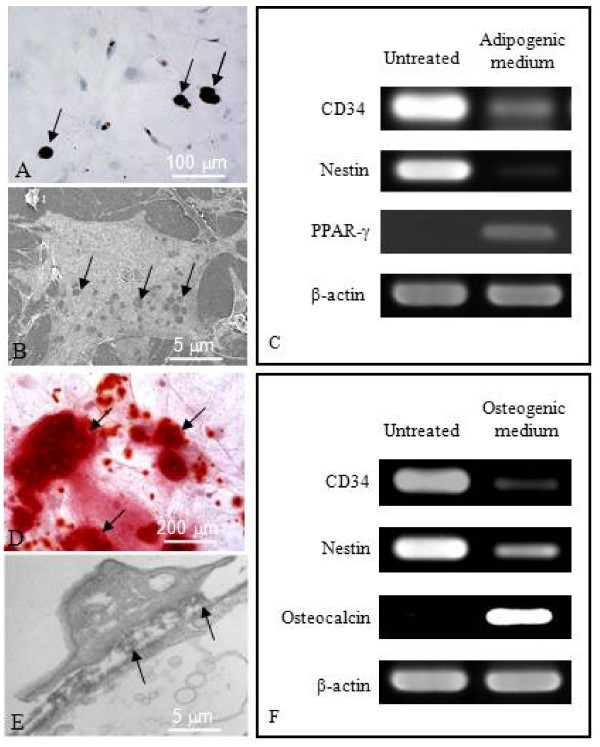
**Developmental potential of HBPCs**. (A) Adipogenic potential of HBPCs was examined. Oil Red O dye staining revealed the presence of adipocytes (arrows) in the HBPC cultures, 3 weeks after adipogenic-inducing medium treatment. Approximately 27 ± 3% of the induced cells were adipocytes. (B) Scanning electron micrograph showing a representative adipocyte that have formed in the cultures. The cytoplasm of these cells was filled with numerous small lipid vesicles (arrows). (C) Semi-quantitative RT-PCR analysis revealed that after the HBPCs were incubated with adipogenic-inducing medium for 3 weeks, CD34 and Nestin (HBPC marker) expression were inhibited while PPAR-γ (adipocyte marker) expression was induced. β-actin served as an internal control. (D) Osteogenic potential of HBPCs was also examined. Alizarin Red S staining revealed the presence of osteocytes (arrows) in the cultures, after 3 weeks osteogenic-inducing medium induction. Approximately 31 ± 3% of the treated cells have transdifferentiated into osteocytes (arrows). (E) Transmission electron micrograph showing a representative osteocyte that has transdifferentiated from the HBPCs. These cells were capable of secreting bone matrix (arrows). (F) Semi-quantitative RT-PCR analysis revealed that after HBPCs were incubated in osteogenic-inducing medium for 3 weeks, CD34 and Nestin expressions were inhibited while Osteocalcin (bone marker) was activated. β-actin served as an internal control.

The multipotency of HBPCs was assessed for their ability to transdifferentiate into adipocytes and osteocytes. The HBPCs were cultured in the presence of adipogenic or osteogenic-inducing media. We established that the HBPCs could be readily induced to differentiate into adipocytes after culturing 21 days that they were positively stained with Oil Red O solution (Figure 3A). Under scanning electron microscopy, the cytoplasm of induced HBPCs clearly show the presence of empty vacuoles which originally contained storage of lipids (Figure 3B). Semi-quantitative RT-PCR analysis revealed that, following adipogenic-inducing medium treatment, CD34 and Nestin (HBPC markers) were down-regulated whereas PPAR-γ (adipocyte marker) expression was up-regulated (Figure 3C). Similarly, HBPCs could be induced to transdifferentiate into osteocytes by osteogenic-inducing medium (Figure 3D). Transmission electron microscopy revealed that the induced HBPCs could secrete bone matrix-like materials into the interstitial space (Figure 3E). Semi-quantitative RT-PCR analysis showed that CD34 and Nestin expression were down-regulated while osteocalcin (osteocyte marker) expression was up-regulated (Figure 2F).

**Figure 4:**
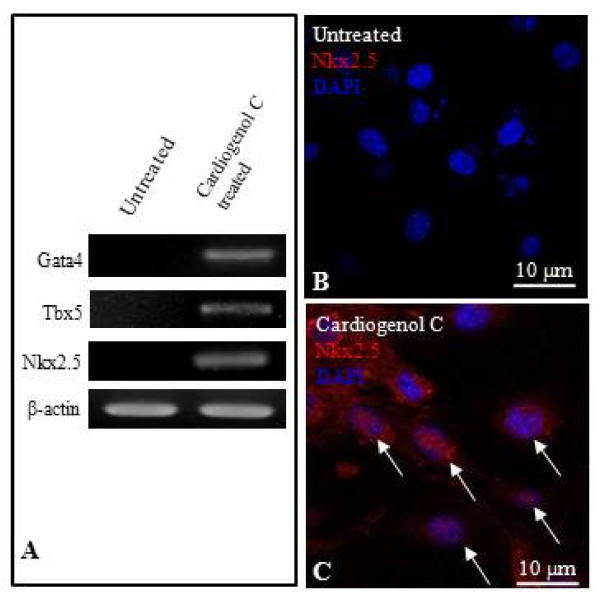
**Cardiomyogenic induction of HBPCs by Cardiogenol C**. (A) Semi-quantitative RT-PCR analysis revealed that Cardiogenol C could induce HBPCs to express cardiac-specific markers GATA4, Tbx5 and Nkx2.5. β-actin served as an internal control. (B-C) Immunofluorescent staining showed HBPCs expressed myogenic transcription factor Nkx2.5 (white arrows) following 5 μM Cardiogenol C treatment for 4 days. In contrast, the untreated samples were negatively stained.

**Figure 5:**
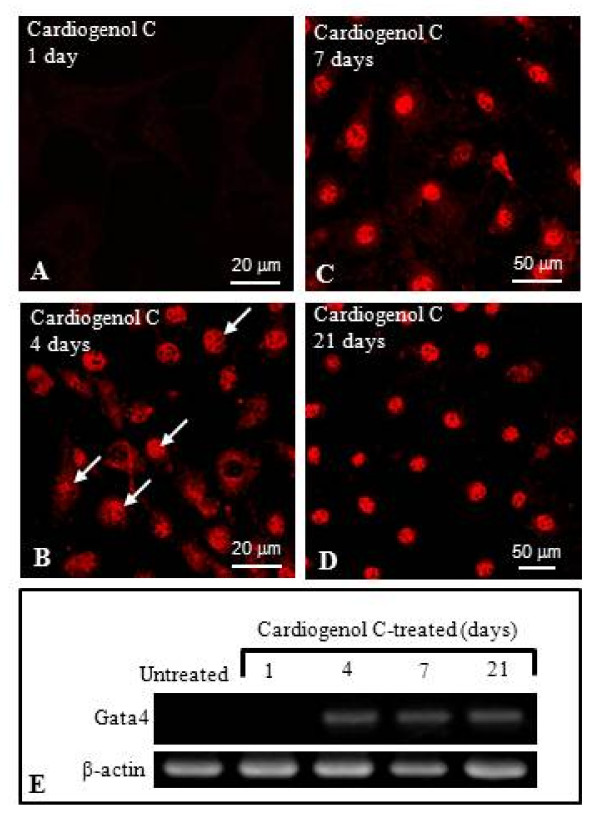
**Cardiogenol C induces HBPCs to express GATA4**. Immunofluorescent staining (A-D) and semi-quantitative RT-PCR analysis (E) revealed that HBPCs began expressing GATA4 (arrows) 4 days after Cardiogenol C treatment.

**Figure 6:**
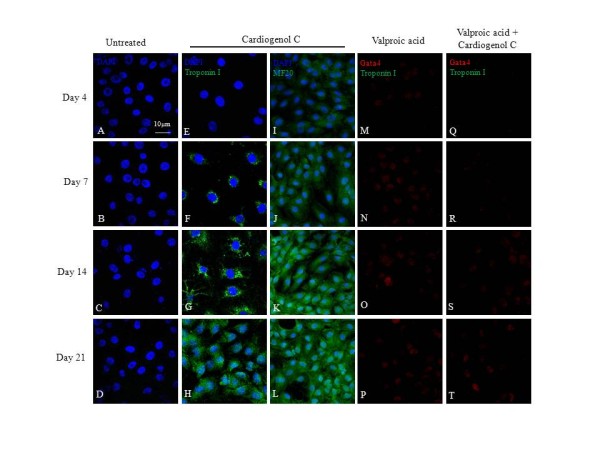
**Effects of Valproic acid on cardiomyogenic induction by Cardiogenol C**. Cardiogenol C-treated and untreated HBPCs were stained with Cardiac Specific Troponin I and sarcomeric myosin heavy chain (MF20) antibodies. The results revealed that Cardiogenol C-treated HBPCs expressed cardiac specific troponin I (G-H) and sarcomeric myosin heavy chain (J-L) progressively. HBPCs treated with Valproic acid alone (M-P) or Valproic acid plus Cardiogenol C (Q-T) did not induce GATA4 or cardiac specific troponin I expressions (M-T).

**Figure 7:**
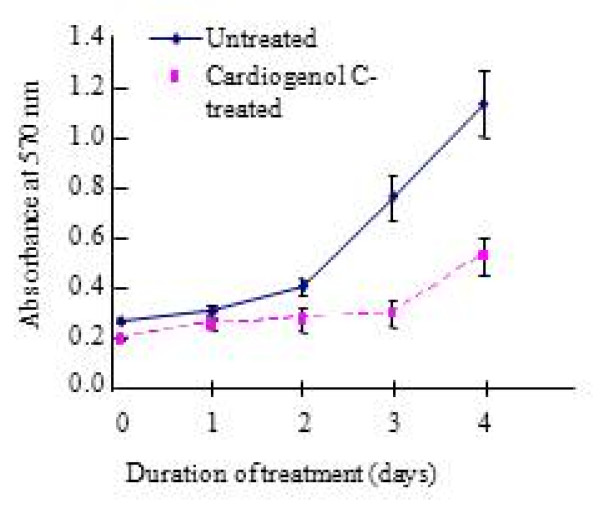
**Effects of Cardiogenol C on HBPCs proliferation**. MTT assay showed that Cardiogenol C treatment significantly inhibited HBPCs proliferation.

We also investigated the ability of HBPCs to transdifferentiate into cardiomyocytes using small molecule, Cardiogenol C. Semi-quantitative RT-PCR analysis revealed that Cardiogenol C could activate the expression of transcription factors GATA4, Tbx5 and homeodomain protein Nkx2.5, which are all early pre-cardiac cell markers that are indispensible for initiating cardiomyogenesis (Figure 4A). Immunofluorescent staining further confirmed that Cardiogenol C induced expressions of cardiac marker Nkx2.5 (Figure 4B) and GATA4 (Figure 5A-D). In addition, western blot analysis revealed that GATA4 expression was initiated from day 4 culture onwards in Cardiogenol C-treated HBPCs (Figure 5E). Immunofluorescent staining showed the Cardiogenol C-treated HBPCs also progressively expressed Cardiac-specific troponin I (Figure 6E-H) and sarcomeric myosin heavy chain proteins (Figure 6I-L). However, we did not observe any contracting cells in the cardiogenol C-treated cultures. In this context, we called these cells cardiomyocyte-like cells rather than cardiomyocytes. Huangfu et al. reported that treating fibroblasts with Valproic acid, a histone deacetylase inhibitor, enabled the fibroblasts to be more efficiently reprogrammed to become induced pluripotent stem cells [22]. Hence, we treated our HBPCs simultaneously with Valproic acid and Cardiogenol C. The mixture did not improve cardiomyocyte transdifferentiation. In fact, the presence of Valporic acid inhibited the process (Figure 6M-T). We also investigated the effects of Cardiogenol C on cell division. MTT assay revealed that Cardiogenol C significantly inhibited cell proliferation (Figure 7).

### Comparative proteomic analysis

**Figure 8:**
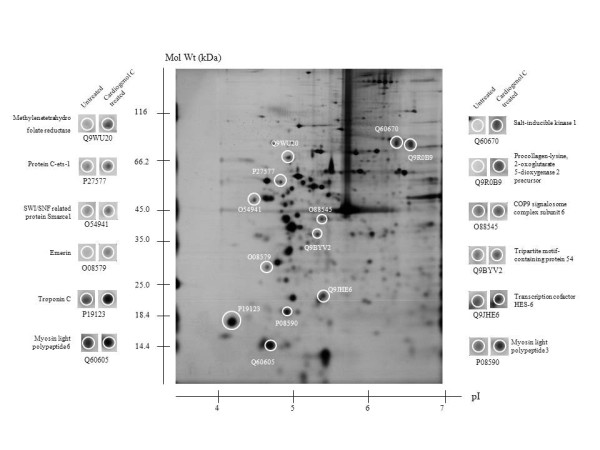
**Comparative proteomic analysis: Identification of protein up-regulated by Cardiogenol C**. The silver stained 2-DE gel showing proteins differentially up-regulated in HBPCs by Cardiogenol C.

**Figure 9:**
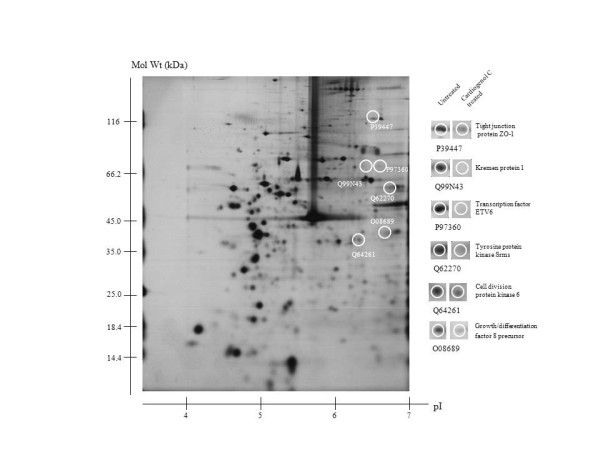
**Comparative proteomic analysis: Identification of protein down-regulated by Cardiogenol C**. The silver stained 2-DE gel showing proteins differentially down-regulated in HBPCs by Cardiogenol C.

We used comparative proteomics to elucidate how Cardiogenol C was able to induce HBPCs to become cardiomyocyte-like cells. Two-dimensional gel electrophoresis was performed and the protein profile of HBPCs treated with Cardiogenol C for four days was compared with untreated HBPCs. We identified 18 silver-stained protein spots that were differentially expressed from 3 independent experiments. Twelve of the proteins were up-regulated by Cardiogenol C treatment (Figure 8), while 6 of the proteins were down-regulated (Figure 9). MALDI-TOF MS analysis revealed that the up-regulated proteins included: 1) COP9 signalosome complex subunit 6, 2) emerin, 3) methylenetetrahydrofolate reductase (Mthfr), 4) myosin light polypeptide 3, 5) myosin light polypeptide 6, 6) procollagen-lysine, 2-oxoglutarate 5-dioxygenase 2 precursor (Plod2), 7) protein C-ets-1, 8) salt-inducible kinase 1 (SIK1), 9) SWI/SNF related protein Smarce1, 10) transcription cofactor HES-6, 11) tripartite motif-containing protein 54, and 12) troponin C (summarized in Table 2). The down-regulated proteins were included: 1) cell division protein kinase 6 (Cdk6), 2) growth/differentiation factor 8 precursor (GDF-8), 3) Kremen protein 1 precursor (Kremen1), 4) tight junction protein ZO-1, 5) transcription factor ETV6, and 6) Tyrosine protein kinase Srms. The observed pI and molecular mass of each proteins identified on the 2DE gel matched closely with the theoretical values provided in the bioinformatic database. Their functions were also summarized in the Table 2 and 3.

**Table 2:** Comparative proteomic analysis: proteins up-regulated by Cardiogenol C in HBPCs

Proteins identified	Observed MW(kDa)/pI	Theoretical MW (kDa)/pI	Accession number	Folds increase	Reported functions
COP9 signalosome complex subunit 6	41/5.4	35.9/5.5	O88545	1.7	A complex involved in various cellular and developmental processes. It is an essential regulator of the ubiquitin conjugation pathway
Emerin	27/4.6	29.4/4.9	O08579	2.3	Emerin may be required for the stability and normal function of rigorously moving nuclei in skeletal muscle and heart
Methylenetetrahydro folate reductase (Mthfr)	70/4.9	74.7/5.2	Q9WU20	2.4	Folate is important for normal heart development. It is a key enzyme in folate and homocysteine metabolism
Myosin light polypeptide 6	14/4.7	16.8/4.6	Q60605	1.4	Regulatory light chain of myosin
Myosin light polypeptide 3	19/4.9	21.8/5.0	P08590	1.6	Regulatory light chain of myosin
Procollagen-lysine,2- oxoglutarate 5- dioxygenase 2 precursor (Plod2)	80/6.5	84.5/6.3	Q9R0B9	4.0	Essential for the stability of the intermolecular collagen cross-links; highly expressed in heart, lung, kidney, eye, ovary, and placenta
Protein C-ets-1	55/4.8	50.2/5.0	P27577	1.7	Transcription factor. Binds to DAXX. Interacts with UBE2I. Essential for normal coronary and myocardial development in chicken embryos
Salt-inducible kinase (SIK1)	81/6.4	85.0/6.4	Q60670	3.8	Transient role during the earliest stages of myocardial cell differentiation and/or primitive chamber formation and may also be important for the earliest stages of skeletal muscle growth and/or differentiation. Potential role in G2/M cell cycle regulation
SWI/SNF related protein Smarce1	49/4.5	46.6/4.9	O54941	1.9	Involved in transcriptional activation and repression of select genes by chromatin remodeling (alteration of DNA-nucleosome topology)
Transcription cofactor HES-6	23/5.3	24.4/5.2	Q9JHE6	1.4	Up-regulate the transcription of ASCL1 and TCF3. Promotes cell differentiation
Tripartite motif- containing protein 54	36/5.	40.3/5.1	Q9BYV2	1.6	It may bind and stabilize microtubule during myotube formation
Troponin C	17/4.2	18.4/4.0	P19123	1.4	Troponin is the central regulatory protein of striated muscle contraction. The binding of calcium to Tn-C abolishes the inhibitory action of Tn on actin filaments

**Table 3:** Comparative proteomic analysis: proteins down-regulated by Cardiogenol C in HBPCs

Proteins identified	Observed MW (kDa)/pI	Theoretical MW (kDa)/pI	Accession number	Folds decrease	Reported functions
Cell division protein kinase 6 (Cdk6)	36/6.2	37.0/6.2	Q64261	1.7	Probably involved in the control of the cell cycle. Interacts with D-type G1 cyclins
Growth/differentiation factor 8 (GDF-8)	39/6.6	42.9/6.6	O08689	2.2	Acts specifically as a negative regulator of skeletal muscle growth
Kremen protein 1 precursor (Kremen1)	63/6.5	51.7/6.7	Q99N43	3.7	Receptor for Dickkopf protein. Cooperation with Dickkopf to block Wnt/beta-catenin signaling
Tight junction protein ZO-1	130/6.4	194.7/6.2	P39447	1.4	The N-terminal may be involved in transducing a signal required for tight junction assembly, while the C-terminal may have specific properties of tight junctions. The alpha domain might be involved in stabilizing junctions
Transcription factor ETV6	60/6.5	56.4/6.8	P97360	3.6	Transcription repressor; binds to the DNA sequence 5' CCGGAAGT-3'
Tyrosine protein kinase Srms	56/6.6	55.8/6.7	Q62270	1.5	May be involved in proliferation or differentiation of keratinocytes in the skin

**Figure 10:**
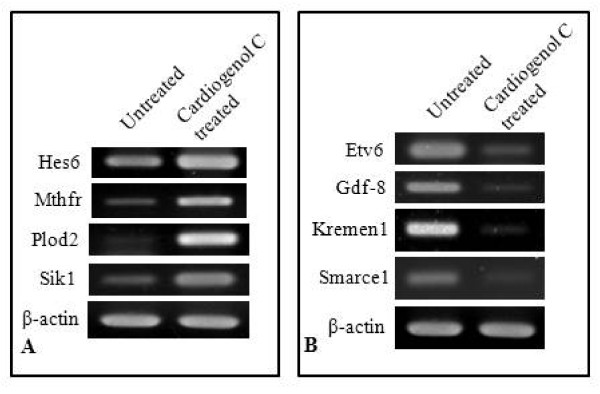
**RT-PCR analysis of differentially expressed proteins**. Semi-quantitative RT-PCR analysis was performed to confirm that some of differentially expressed proteins identified in the comparative proteomics were also differentially transcribed. (A) The transcription levels of Hes6, Mthfr, Plod2 and SIK1 were found up-regulated in the Cardiogenol C-treated samples. (B) ETV6, GDF-8, Kremen1 and Srms expression were down-regulated by Cardiogenol C. β-actin served as an internal control.

We next performed semi-quantitative RT-PCR analysis to determine whether some of the differentially-expressed proteins identified were also affected at the transcriptional level. We established that Hes6, Mthfr, Plod2 and SIK1 transcriptions were up-regulated following Cardiogenol C treatment (Figure 10A); whereas, ETV6, GDF-8, Kremen1 and Srms transcriptions were down-regulated (Figure 10B). These results were the same as those observed in the compare proteomic analyses.

### Cardiogenol C activates Wnt/beta-catenin signaling

**Figure 11:**
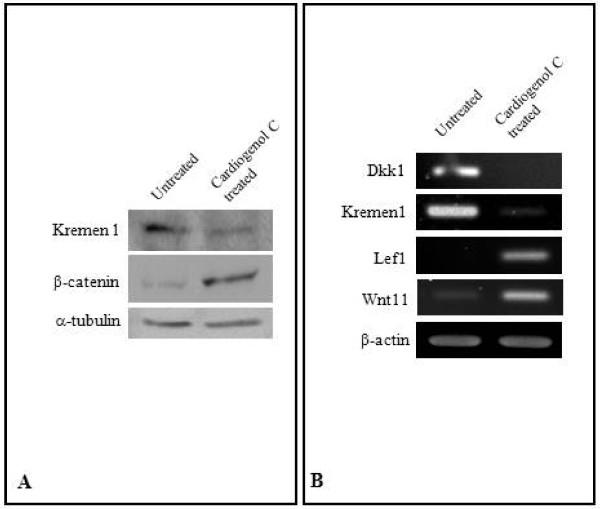
**Activation of Wnt signaling pathway following Cardiogenol C induction**. (A) Western blot analysis revealed that Kremen1 was down-regulated in Cardiogenol C-treated HBPCs. Tubulin served as an internal control. (B) Semi-quantitative RT-PCR analysis revealed Dkk1 and Kremen1, which are normally involved in the Wnt signaling pathway, were down-regulated in Cardiogenol C-treated HBPCs while Lef1 and Wnt11 expression were up-regulated.

**Figure 12:**
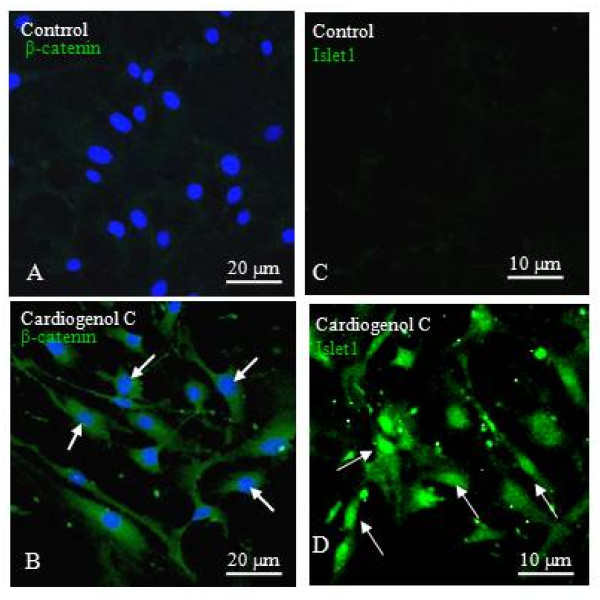
**β-catenin and Islet1 expression in HBPCs after Cardiogenol C treatment**. (A-B) Immunofluorescent staining revealed the presence of the β-catenin (white arrows) in the Cardiogenol C-treated HBPCs but not in the untreated HBPCs. (C-D). Immunofluorescent staining revealed HBPCs expressed Islet1 (white arrows) following Cardiogenol C-treatment but not in the untreated cells.

Kremen1 was one of the proteins found down-regulated in our comparative proteomic analysis. This protein normally acts as a receptor for Dickkopf (DKK) protein and both cooperate together to block Wnt/β-catenin signaling [23,24]. Hence, we decided to investigate whether the presence of Cardiogenol C could activate the Wnt/β-catenin pathway. Western blot analyses revealed that there were significant increase in the Kremen1 and β-catenin following Cardiogenol C treatment (Figure 11A). It has been reported that Wnt-11 is one of the potential activator of the Wnt/β-catenin signaling during cardiogenesis [25]. Transcriptional factor, Lef1, participates in Wnt/β-catenin signaling by mediating in the phosphorylation of β-catenin [26]. We established that Dkk1 and Kremen1 expression were down-regulated; whereas, Lef1 and Wnt11 expression were up-regulated by semi-quantitative RT-PCR analysis (Figure 11B). Immunofluorescent staining revealed that β-catenin was detected in the cytoplasm and nucleus of Cardiogenol C-treated HBPCs at Day 3 but not in untreated cultures (Figure 12A-B). Recently, Islet1 has been reported to be a downstream target of β-catenin in cardiac progenitor cells [27]. Therefore, we examined whether Cardiogenol C could induce HBPCs to express Islet1. We established that the Cardiogenol C-treated cells expressed Islet1 after 3 days culture (Figure 12C-D).

### Cardiogenol C suppresses genes involved in chromatin remodeling

**Figure 13:**
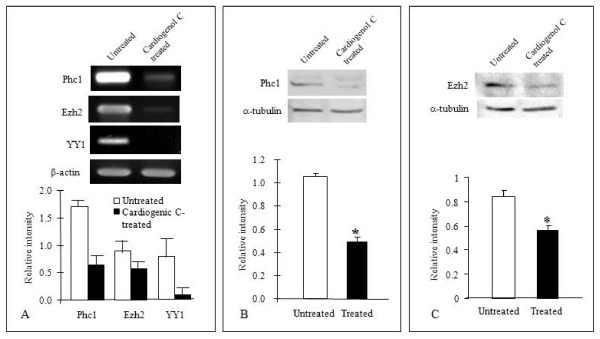
**Cardiogenol C inhibits chromatin remodeling gene expression in HBPCs**. (A) Semi-quantitative RT-PCR analysis of three components of the polycomb gene complex (Phc1, Ezh2 and YY1). Expressions of all three genes were down-regulated in HBPCs following Cardiogenol C-treatment. The bar chart showed the normalized density measurements of the PCR bands. (B and C) Western blot analyses confirmed that Cardiogenol C inhibited Phc1and Ezh2 expressions in HBPCs. α-tubulin served as an internal control. The bar charts showed the normalized density measurements of the protein bands. The experiments were performed in triplicates, and each data point was expressed as mean value ± SEM. *Statistically significant difference (p < 0.05).

SIK1 was also one of the proteins that we found up-regulated in the comparative proteomic analysis. SIK1 has been identified as a class II Histone deactylases (HDACs) kinase that is specifically expressed in the mouse embryonic heart [28]. SIK1 is known to phosphorylate cytoplasmic class II HDACs to trigger their translocation into the nucleus and activate MEF2-dependent transcription [28,29]. This suggests that chromatin remodeling is also involved in Cardiogenol C-induced cardiogenesis. Recent studies revealed that the Polycomb gene complex may competitively antagonize nucleosome remodeling by the SWI/SNF-family complex [30]. Hence, we examined the effects of Cardiogenol C on the polycomb group gene complex. Semi-quantitative RT-PCR analysis revealed that polyhomeotic-like 1 (Phc1), Zeste homolog 2 (Ezh2) and transcription factor YY1 expression were significantly down-regulated following Cardiogenol C treatment (Figure 13A). Moreover, western blot analysis confirmed that Phc1 and Ezh2 expressions were inhibited by Cardiogenol C (Figure 13B-C).

## Discussion

Previous studies on HBPCs have mostly been related to hair regeneration and re-epithelialisation of burn wound, chronic wound and ulcerated skins [4,31-34]. In the present study, we have demonstrated that the HBPCs, isolated from mouse vibrissa, are multipotent and can potentially provide a source of autologous progenitor cells for cardiac repair. These HBPCs expressed K15, a specific marker for hair bulge stem cells, and also expressed neural crest stem cell markers Nestin and Snail [32]. Furthermore, these cells expressed cell surface markers K5, K14 and CD34 which confirm these cells were originated from the bulge region and not from adjacent connective tissue which do not express these markers [35]. Our HBPCs also expressed Sox2 which is a key transcription factor involved in maintaining pluripotency and self-renewal in embryonic stem cells [36-38]. Since HBPCs express the pluripotent marker Sox2, we investigated the developmental potential of these cells. These cells were able to transdifferentiate into adipocytes and osteocytes when chemically induced. To investigate the ability of HBPCs to transdifferentiate into cardiac cells, we used a small cell permeable molecule called Cardiogenol C. This molecule was first reported to be able to induce embryonic stem cells to differentiate into beating cardiomyocytes [18]. We found that Cardiogenol C-treated HBPCs can be induced to express Nkx2.5 and GATA4, two early markers for precardiac cells [39]. These genes are evolutionary highly conserved and indispensable for normal heart development [39,40]. In mature Cardiogenol C-treated cultures, we established that the cells can also express cardiac-specific troponin I and sarcomeric myosin heavy chain. In contrast to findings reported by Wu et al., who observed beating cardiomyocytes following Cardiogenol C-treated of embryonic stem cells, we could not find cardiomyocytes capable of contracting in our Cardiogenol C-treated HBPCs [18]. In this context, Cardiogenol C cannot be used to produce fully functional cardiomyocytes by HBPCs - despite its ability to induce expression of key cardiac transcriptional factors Nkx2.5, GATA4, Tbx5 and Islet1.

Recently, Huangfu et al. revealed that Valporic acid (a histone deacetylase inhibitor) could be used to enhance the reprogramming of somatic cells into induced pluripotent stem cells by more than 100-fold [41]. We therefore decided to use Valporic acid, in combination with our Cardiogenol C, to induce a more comprehensive transdifferentiation of our HBPCs - producing cardiomycytes that were capable of spontaneous contraction. However, we found that the HBPCs were not responsive to the Valporic acid treatment. Our results imply that HBPCs are only capable of transdifferenting into cardiomyocyte-like cells when induced by Cardiogenol C. We believe that this limited response may be attributed to the developmental plasticity of our HBPCs verses embryonic stem cells (i.e. multipotent verses pluripotent, respectively). Liu et al. recently reported that hair follicle stem cells from the bulge region could differentiate into smooth contractile muscle cells using a tissue-specific promoter [42]. In this study, our isolated CD34^+ ^HBPCs behave like mesenchymal stem cells capable of differentiating into various mesenchymal lineages, such as adipocytes and osteocytes. Though HBPCs can only transdifferentiate into cardiomyocyte-like cells, they may still be potentially useful once a method for stimulating these cells to contract has been established.

In this study, we used comparative proteomic approach to elucidate how Cardiogenol C was able to induce HBPCs to transdifferentiate into cardiomyocyte-like cells. We found a number of differentially expressed proteins in our treated HBPCs. Kremen1 expression was significantly down-regulated in the Cardiogenol C-treated cells. It has been reported that Kremen1 and Kremen2 are two dickkopf homolog 1 (DKK1) transmembrane receptors which regulate the canonical Wnt/β-catenin signaling pathway. The binding of DKK1 to the Kremen receptors antagonize the canonical Wnt/β-catenin signaling by blocking Wnt co-receptors LRP5/6 [23,24]. Both canonical and noncanoncial Wnt signaling pathways are essential regulators for coordinating cardiac specification and morphogenesis. Canonical Wnt/β-catenin signaling regulates early cardiogenesis by enhancing the proliferation of cardiac progenitors and differentiation of cardiomyocytes [43,44]. β-catenin is thought to interact with members of the LEF-1/TCF family of transcription factors to mediate in Wnt signaling [26]. β-catenin also modulates the expression of Islet1 in cardiac progenitor cells which is required for cardiogenesis [27]. The noncanonical Wnt signaling pathway, which is independent of β-catenins, involves protein kinase C and Jun amino-terminal kinase also regulates cardiac differentiation [45]. Wnt11 in the noncanonical pathway was reported to enhance cardiomyocytes differentiation in various stem cell populations [25,46,47]. In our semi-quantitative RT-PCR studies, we found Lef1 and Wnt11 expression were up-regulated by Cardiogenol C. Furthermore, our immunofluorescent staining results revealed that β-catenin was present in both the nucleus and cytoplasm. Therefore, it appears that Cardiogenol C could activate Wnt/β-catenin signaling to induce cardiogenesis. The results of our MTT cell proliferation assay confirmed that Cardiogenol C-treatment significantly decreased HBPCs proliferation. Nevertheless, we cannot explain why Cardiogenol C induced an increase in β-catenin yet a decrease in cell proliferation, as activation of the Wnt signaling pathway is normally associated with increased cell proliferation. This paradox may be required to be investigated in the future.

Besides cardiac-inducing transcription factors, epigenetic factors may also play a contributory role in cardiomyocyte differentiation. This idea is supported by reported findings that 5-azacytidine, an unspecific DNA methyltransferase inhibitor, can induce cardiogenesis [48]. This reagent prevents methylation at cytosine, which makes CpG islands in the promoter sequences of genes involved in cardiac differentiation. The unmethylated sequence allows the binding of transcription initiation machinery. Moreover, several chromatin remodeling proteins, such as methyltransferase Smyd1, SWI/SNF protein Baf60c, HDAC5 and HDAC9, have also been implemented in cardiomyocytes differentiation [49-51]. In this context, we identified two chromatin remodeling proteins, SIK1 and Smarce1, which were up-regulated by Cardiogenol C in our comparative proteomic analysis. SIK1 is a kinase of class II HDACs. It stimulates cardiac-specific transcription factor Mef2 via phosphorylation of HDACs [29]. Smarce1 is a component of the SWI/SNF complex. It can interact specifically with transcription factor REST to repress neuronal genes.

Therefore, up-regulation of Smarce1 might facilitate the repression of neuronal- and neural crest-related genes (e.g. Nestin and Snail) in our Cardiogenol C-treated HBPCs. Recently, the polycomb group complex proteins have been identified as essential in the maintenance of embryonic and adult stem cells, by silencing genes that are necessary for stem/progenitor cells to differentiate into various tissue types [52-54]. Therefore, we examined whether the polycomb group proteins were also involved in cardiac differentiation induced by Cardiogenol C. We found that Cardiogenol C suppressed Phc1, Ezh2 as well as YY1 expression. Ezh2 contains SET domain and belongs to polycomb repressor complex 2; while Phc1 and YY1 contain zinc-finger domain and are components of PRC1 maintenance complex [54,55]. These findings lead us to speculate that up-regulation of SIK1 as well as down-regulation of polycomb group proteins may silence genes that normally represses cardiac differentiation.

We have also identified several more proteins that were down-regulated by Cardiogenol C. Cdk6 was inhibited by Cardiogenol C. This protein is a vertebrate cdc-2 related kinase. It interacts with the G-type cyclins in the early G1 phase and functions as a retinoblastoma protein (Rb) kinase that phosphorylates the Rb protein. Phosphorylated Rb releases its binding partner transcription activator E2F. The free E2F in turn stimulates the transcription of genes essential for DNA replication, which initiates the cell cycle into the S phase [56]. Indeed, it has also been reported that cdk6 expression must be suppressed in order to allow proper osteoblasts and osteoclasts differentiation [55,57,58]. Therefore, it would be expected that mitogenic cdk6 expression would be inhibited so that the HBPCs could exit the cell cycle to initiate differentiation. Myostatin (also known as GDF-8) expression was also suppressed in response to Cardiogenol C treatment. Morissette et al. reported that myostatin was a negative regulator involved in controlling the growth of striated muscles in the heart [59]. Therefore, it was not surprising to observe the decreased myostatin expression when Cardiogenol C-treated HBPCs transdifferentiate into cardiomyocyte-like cells.

In conclusion, we demonstrated for the first time that HBPCs can be induced to transdifferentiate into cardiomyocyte-like cells using Cardiogenol C. With more research into understanding the developmental properties of HBPCs, these readily accessible cells may in the future provide an abundant potential source of progenitor cells for the therapeutic treatment of heart diseases.

## Authors' contributions

WWWY conducted the Cardiogenol C studies and the draft of the manuscript; MKT performed comparative proteomics analysis, histology and also help prepare the manuscript; EC performed the RT semi-quantitative assay, MTT assay; YY carried out the western blot assay; I WC isolated and characterized the mouse hair bulge progenitor cells, comparative proteomics, SEM and TEM studies; HSSL performed the immunohistological staining and KKH L designed and coordinated the experiments, proof read the manuscript. All authors read and approved the final manuscript.

All of the authors declare that they have no competing interests.
